# Ultrasound-Targeted Microbubble Destruction: Modulation in the Tumor Microenvironment and Application in Tumor Immunotherapy

**DOI:** 10.3389/fimmu.2022.937344

**Published:** 2022-07-01

**Authors:** Ye Han, Jiawei Sun, Hong Wei, Jiarong Hao, Weiyao Liu, Xiaolei Wang

**Affiliations:** In-Patient Ultrasound Department, Second Affiliated Hospital of Harbin Medical University, Harbin, China

**Keywords:** ultrasound-targeted microbubble destruction, tumor microenvironment, tumor angiogenesis, ultrasonic cavitation, tumor immunotherapy, endothelial cells

## Abstract

Tumor immunotherapy has shown strong therapeutic potential for stimulating or reconstructing the immune system to control and kill tumor cells. It is a promising and effective anti-cancer treatment besides surgery, radiotherapy and chemotherapy. Presently, some immunotherapy methods have been approved for clinical application, and numerous others have demonstrated promising *in vitro* results and have entered clinical trial stages. Although immunotherapy has exhibited encouraging results in various cancer types, however, a large proportion of patients are limited from these benefits due to specific characteristics of the tumor microenvironment such as hypoxia, tumor vascular malformation and immune escape, and current limitations of immunotherapy such as off-target toxicity, insufficient drug penetration and accumulation and immune cell dysfunction. Ultrasound-target microbubble destruction (UTMD) treatment can help reduce immunotherapy-related adverse events. Using the ultrasonic cavitation effect of microstreaming, microjets and free radicals, UTMD can cause a series of changes in vascular endothelial cells, such as enhancing endothelial cells’ permeability, increasing intracellular calcium levels, regulating gene expression, and stimulating nitric oxide synthase activities. These effects have been shown to promote drug penetration, enhance blood perfusion, increase drug delivery and induce tumor cell death. UTMD, in combination with immunotherapy, has been used to treat melanoma, non-small cell lung cancer, bladder cancer, and ovarian cancer. In this review, we summarized the effects of UTMD on tumor angiogenesis and immune microenvironment, and discussed the application and progress of UTMD in tumor immunotherapy.

## Introduction

In the past decade, rapid advancements in tumor immunotherapy have established it as a crucial treatment for various kinds of cancers (1). Compared with surgery, radiotherapy and chemotherapy that directly act on the tumor itself, tumor immunotherapy stimulates the body’s immune system and indirectly attacks tumor cells by enhancing the immune defense mechanism against the tumor and reshaping the immune microenvironment (2, 3). On the one hand, it can enhance immune-mediated tumor cell death by promoting immune tumor cell recognition and eliminating target cells that carry tumor antigens, while on the other hand, it can eliminate or reduce immunosuppressive signals induced by tumor cells (4, 5).

At present, the common tumor immunotherapy includes tumor vaccines, tumor-agnostic therapies, gene therapies and adoptive cell immunotherapies. Nano-based drug delivery systems (6) and cell-inspired drug delivery platforms (7) are also being used in cancer immunotherapy. A variety of immunotherapy drugs have been approved for clinical use and are benefiting patients with lung cancer (8), bladder cancer (NEO-PV-01) (8), melanoma (NeoVax) (9), and ovarian cancer (OCDC) (10). However, some patients have poor responses to immunotherapy and may even develop hyper progressive disease after treatment. Positive responses to immunotherapy usually depend on the dynamic interactions between tumor cells and immunomodulators in the tumor microenvironment. Low immune responses are often associated with tumor angiogenesis and tumor-specific immunosuppressive microenvironments (11). In addition, complexities in the structures and functions of tumor angiogenesis make drug penetration very challenging, resulting in insufficient drug delivery (12).

**Figure 1:**
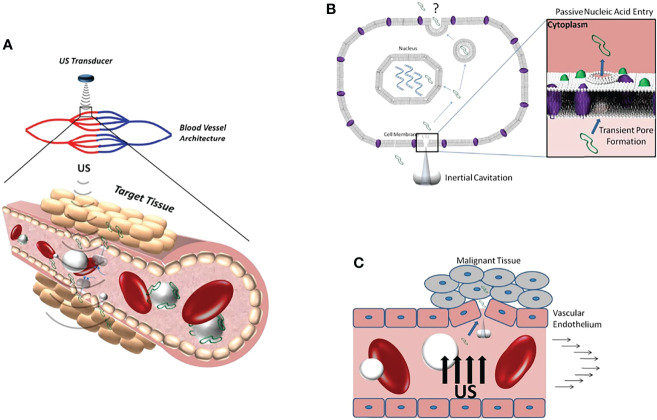
**(A)** Schematic diagram of ultrasonic cavitation promoting DNA (green) extravasation into tissue. **(B)** Microjets generated by inertial cavitation creates acoustic pores that allow DNA to enter the cytoplasm. **(C)** Inertial cavitation increases the permeability of blood vessels to DNA by damaging the integrity of the vascular endothelium. Reprinted with permission from ref (13). copyright ^©^ 2012 Sirsi and Borden.

Ultrasound-targeted microbubble destruction (UTMD) utilizes microstreaming, microjets and free radicals generated by ultrasonic cavitation to damage endothelial cells (ECs) (**Figure 1**). Similar to sonodynamic therapy, this regulates the tumor immunosuppressive microenvironment by causing microvascular rupture and tumor cell apoptosis, hindering tumor angiogenesis, and enhancing immunotherapy effects (14–16). Further, local ultrasound irradiation can trigger the targeted release of drugs and exogenous genes to achieve higher treatment efficiency (17). Therefore, UTMD has shown promising prospects in improving the therapeutic efficacies of immunotherapy.

In this article, we summarized the regulation of UTMD on tumor angiogenesis and the immune microenvironment. Then, we reviewed the practical application of UTMD in various tumor immunotherapies, including tumor vaccines antibody therapy, gene therapy and adoptive cell therapy, and their projected future developments (**Table 1**).

**Table 1:** Application of UTMD combined with immunotherapy in various cancers.

Immunotherapy methods	Cancer types	Treatment	Animal model	Outcomes	References
Antibody immunotherapy	Prostate cancer+melanoma+colon cancer	USNBs+anti-PD-L1	Female C57BL/6 and nude mice	Promote the infiltration and antitumor activity of CD8+ T cells, increase DAMP release and tumor antigen presentation	(18)
Antibody immunotherapy	HER2-positive breast cancer brain metastasis	FUS+circulating MBs+trastuzumab	Male nude rats	Decrease tumor volume and improve survival	(19)
Antibody immunotherapy	HER2-positive gastric cancer	UTMD+sonosensitizer+trastuzumab	Female nude mice	Inhibit the tumor growth	(20)
Antibody immunotherapy	Glioma	UTMD+anti-PD-L1	Female Cr. NIH Swiss mice	improve the penetration depth and transmission efficiency of anti-PD-L1	(21)
Tumor vaccine	Melanoma lung metastasis	UTMD+model antigen (ovalbumin)	C57BL/6 mice	a four-fold decrease in the frequency of melanoma lung metastasis	(22)
Tumor vaccine	Melanoma lung metastasis	UTMD+model antigen (ovalbumin)	C57BL/6 mice	Active exogenous antigen-specific CTL	(23)
Gene therapy	Breast cancer	UTMD+anti-PD-L1+pGM-CSF	Female FVB mice	Increase the plasmid transfection rate and gene expression	(24)
Gene therapy	Hepatocellular carcinoma	UTMD+pre-miRNA plasmids	Male BALB/c nude mice	Suppress the tumor growth	(25)
Gene therapy	Metastatic mammary carcinoma	UTMD+pIFN-β+anti-PD-L1	Female FVB mice	Enhance T cell infiltration and reduce tumor volume	(26)
Adoptive cell immunotherapy	Breast cancer brain metastasis	UTMD+NK-92 cells	Male athymic nude mice	Decrease tumor volume and improve survival	(27)
Adoptive cell immunotherapy	Colorectal adenocarcinoma	UTMD+Fe-NK cells	NSG female mice	NK cells homing to tumor regions	(28)

Ultrasound-stimulated nanobubbles (USNBs); focused ultrasound (FUS); cytotoxic T lymphocytes (CTL); granulocyte-macrophage colony stimulating factor plasmids (pGM-CSF); miRNA overexpression vectors (pre-miRNA); plasmid encoding IFN-β (pIFN-β); natural killer (NK).

## Tumor Angiogenesis and Immune Microenvironment

The tumor immune microenvironment has the following characteristics (29): (a) contains immune cells that lack antigenic stimulation, which could lead to ineffective inhibition of tumor growth and promote tumor immune escape; (b) decreased proliferative ability and insufficiency of immune cells to infiltrate tumoral tissues due to increased interstitial pressure caused by the tumor that acts as a physical barrier; (c) depletion or transient activation of antigen-specific T cells that cannot effectively inhibit tumor growth; (d) poor release of tumor antigens to peripheral lymph nodes, resulting in inadequate direct or indirect antigen presentation and insufficient T cell initiation, and; (e) failure to recognize and present tumor antigens due to the secretion of a variety of negative immune regulatory factors by tumor cells and immunosuppressive cells, leading to immune escape. All these immunosuppressive microenvironments characteristics contributed to the clinically observed drug resistance and off-target toxicity of immunotherapy.

Solid aggressive malignant tumors grow rapidly by inducing the release of various pro-angiogenic and anti-angiogenic factors that prompt the formations of tumor blood vessels to ensure their survival. Among them, the vascular endothelial growth factor (VEGF) has been found to have the most significant pro-angiogenic effect. It can promote vascular ECs division and support ECs migration for constructing more new vessels (30). In addition, VEGF can also induce the expression of adhesion molecules on ECs, mobilize bone marrow-derived cells, and directly or indirectly promote tumor angiogenesis.

Tumor-induced angiogenesis is characterized by structurally distorted tumor vessels and pericellular insufficiency (31). It can cause severe microenvironmental hypoxia, promote VEGF expression and induce vascular malformations (32), resulting in uneven distribution of blood flow and affecting drug penetration and delivery (12). Further, blood leakage from malformed vessels can increase interstitial pressure, which reduces the proliferation, infiltration and survival ability of immune cells. These hinder infiltration and lead to senescence and exhaustion and poor tumor-killing ability of immune cells (33).

## Overview on UTMD

As a simple, safe and non-invasive method, ultrasound has been widely used to diagnose and treat diseases. UTMD is a recently developed technology that can target the release of drugs and exogenous genes by augmenting ultrasonic cavitation effects, which has the advantages of being precise, highly efficient and safe, with good repeatability (34–36). The cavitation effect is a significant physical impact of ultrasound. When ultrasonic pressure reaches a certain threshold, the surrounding liquid is rapidly filled with small cavities of gas and steam, forming microbubbles (MBs), also known as cavitation nuclei. Under the activity of ultrasound, these MBs continue to vibrate, expand and contract, which, when finally burst and collapse (37, 38), release instantaneous energy and cause extreme physical phenomena such as luminescence, high temperature, high pressure, discharge, and microjet (39).

Cavitation effects can be divided into non-inertial cavitation (i.e., stable cavitation) and inertial cavitation (i.e., unstable cavitation) (40). When the ultrasonic amplitude is low, the bubbles can oscillate symmetrically around an equilibrium radius without bursting under the action of ultrasonic, producing microflows characterized by fluid flow (41). Microflows impose shear stress on cells while generating heat and lead to sound holes, which help open the tissue barrier formed by ECs. When the ultrasonic amplitude is large, the bubbles oscillate asymmetrically, their volume expands asymmetrically and collapses. The intense compression of gasses inside the bubbles and the huge fluctuation of local pressure generated by the surrounding fluid are called shock waves, which have substantial impacts on cells or tissues and can locally produce high temperature and high pressure. The energy generated by bubble collapse is then converted to kinetic energy, which allows the fluid to be ejected and leads to irreversible tissue or cell damage (42, 43). This significantly increases the permeability of the tumors’ cell membrane, causing damage and widening the gap of ECs, and DNA breakage, which eventually leads to microvascular rupture, hemorrhage and hemolysis (44). MBs can implode under certain acoustic pressure irradiation, which significantly increases the number of cavitation nuclei and enhances the cavitation effect. The fundamentals of cavitation effects are as follows (45): (a) exogenous MBs increase the number of cavitation nuclei, which then increase the intensity of the cavitation effects; (b) as the quantity of MBs increases, the energy required to produce cavitation decreases and the energy threshold for cavitation effect decreases. Moreover, immunotherapy drugs, exogenous genes, or acoustic sensitizers can be incorporated into MBs to target specific tissues. Ultrasound can irradiate the target tissue with a certain amount of radiation energy, destroying the MBs carrying the drugs and releasing the payload, thus achieving the targeted release of drugs or genes (46). Under the action of low-frequency ultrasound, the MB collapse process caused by the cavitation effect produces jet and releases energy, which can instantly break adjacent cell membranes, increasing their permeability and promoting the phagocytosis of the cell to drugs (47). Furthermore, UTMD can temporarily allow immunotherapy drugs to cross the blood-brain barrier (BBB) and blood-tumor barrier and reach the targeted tumor area (48). Therefore, UTMD-mediated antitumor drug release can reduce the off-target toxicity of tumor immunotherapy. As of now, many studies have utilized UTMD to enhance the efficiency of drug targeting and delivery in local tissues (49, 50).

## Modulation of UTMD on Tumor Angiogenesis

### Antitumor Angiogenesis

Microstreaming and microjets from ultrasonic cavitation-related biological effects can cause ECs damage and microvascular rupture (51). Due to the rapid, loose, and irregular growth of tumor blood vessels and functional defects in their vascular architecture, UTMD can cause significant damage to tumor vascular ECs, manifesting as endothelial cell malformations or endothelial cell contractions (52). Under appropriate sound pressure, UTMD can damage tumor vascularization and exert its antitumor angiogenesis effects (53).

Liu et al. (54) used UTMD (acoustic pressure: 2.6 MPa and 4.8 MPa) to mechanically destroy tumor blood vessels in Walker 256 tumors. They found that contrast-enhanced ultrasound could disrupt tumor neovasculature and significantly decrease tumor perfusion compared with the control group. Histopathologically, the tumor microvascular were destroyed into diffuse hematomas. In a study by Jing et al. (55), the authors showed that the microcirculation of Walker 256 tumors treated with 4.8 MPa could be blocked for 24 h. In a previous study, the investigators used lipid shell MBs loaded with Endostar combined with UTMD to explore the anti-angiogenesis effect of UTMD in established nude mice breast cancer models. Compared with the Endostar group alone, they observed that after ultrasound targeted irradiation of drug-loaded MBs, the release of Endostar was significantly increased, and tumor VEGF expression was significantly down-regulated. Tumor growth inhibition rate was significantly increased, confirming that UTMD combined with drug-carrying MBs could improve the anti-angiogenesis effect of Endostar by downregulating VEGF expression, thus, achieving tumor growth inhibition. Meanwhile, UTMD can release targeted drugs that can accumulate in tumors. Yu et al. (56) treated rats inoculated with Walker 256 tumors using Endostar combined with UTMD and measured the microvascular density by contrast-enhanced ultrasound. They observed that UTMD could significantly lower tumor blood perfusion and had a significantly higher tumor growth inhibition rate than the control group, thus confirming that UTMD enhanced the anti-angiogenic effect of Endostar.

The potential of UTMD in anti-angiogenic therapy remains largely unknown. UTMD at higher energy intensity has been shown to promote apoptosis of ECs by regulating gene expression and contributing to microvascular destruction. Su et al. (57) demonstrated that UTMD (0.5 MHz, 210 mW/cm2) significantly promoted apoptosis and inhibited the angiogenesis of human umbilical vein ECs and human microvascular ECs through the phosphorylation of p38 mitogen-activated protein kinase and activated endoplasmic reticulum stress signal. These results demonstrate the potential value of UTMD in anti-angiogenic therapy.

### Enhanced ECs Permeability

Tumor vascular ECs are the first contact point of cavitation effects (58). UTMD was shown to enhance endothelial cell permeability in *in vivo* and *in vitro* settings, reversibly opening the BBB or blood-tumor barrier and facilitating extracellular drug transfer into the interstitial space. Hallow et al. (59) quantified the biological effects of UTMD on ECs using isolated live pig carotid arteries. Their results showed that relatively low ultrasound energy (700 kPa-1400 kPa) could target 9%-24% of the drug uptake of ECs. Lelu et al. (60) compared the effects of inertial and non-inertial cavitation on the monolayer resistance and permeability of pigs brain’s ECs in the presence of SonoVue. Their results demonstrated that non-inertial cavitation had better cell permeability than inertial cavitation, could reversibly open the BBB and promoted drug delivery. Wang et al. (61) showed that gambogic acid-loaded porous-lipid MBs in combination with UTMD could instantly increase BBB permeability and promote the release of gambogic acid into the stroma of human glioma (U251 cell line), and could also significantly inhibit the tumor’s growth in *in vitro* BBB model of mouse brain endothelial cell line. UTMD has also shown therapeutic potential in pancreatic cancer mouse models. In a study by Zhang et al. (62), the authors showed that UTMD enhanced the permeability of the hematoma barrier through cavitation effects. This promoted the delivery of drug-loaded MBs to the tumor matrix and inhibited the growth rate of pancreatic cancer by 89.8% during 21 days of treatment. Zhang et al. (63) utilized C6 glioma-bearing rats to study the mechanism of UTMD in improving BBB permeability. They observed that the enhanced BBB permeability could be associated with the downregulation of cellular junctional adhesion molecule-A and up-regulation of calcium-activated potassium channel expression, which affected the BBB tight connection.

It was shown that intermittent ultrasound irradiation, compared with continuous ultrasound irradiation, improved the permeability of BBB and promoted the extravasation of Evans Blue into the stromal tissues of C6 glioma membranes. Wang et al. (64) confirmed that microRNA-34a encapsulated with nanoparticles combined with UTMD exerted a significant inhibitory effect on castration-resistant prostate cancer by improving membrane permeability and capillary space and promoting the delivery of nanoparticles to prostate cancer xenograft.

## Modulation of UTMD on Tumor Immune Microenvironment

### Damage-Associated Molecular Pattern (DAMP) and Tumor Antigen Presentation

**Figure 2:**
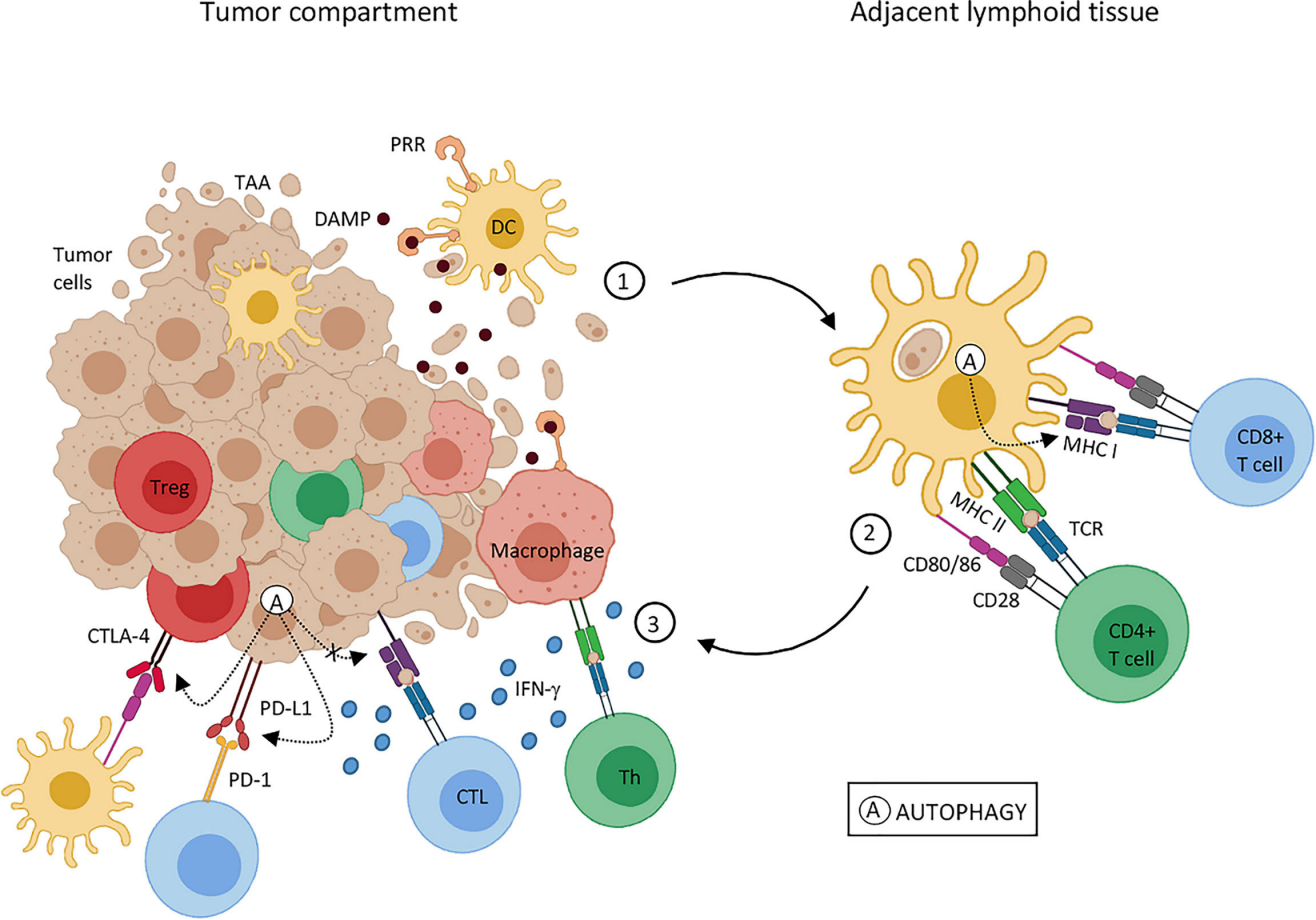
Effects of tumor cell death on tumor-associated antigen presentation. Tumor-associated antigens (TAA); dendritic cells (DC); major histocompatibility complex (MHC); damage-associated molecular pattern (DAMP); cytotoxic T lymphocytes (CTL); T regulatory cells (Treg); pattern recognition receptor (PRR); T-cell receptor (TCR); helper T cell (Th); tumor necrosis factor (TNF); programmed cell death protein 1 (PD-1); programmed cell death-ligand 1 (PD-L1). Reprinted with permission from ref (69). copyright ^©^ 2020 de Souza, Gonçalves, Lepique and de Araujo-Souza.

UTMD promotes tumor cell death by regulating calcium levels and ceramide signaling pathways. Dying or stressed tumor cells release DAMPs, which act as adjuvants or immune recognition stimulants, which trigger immune responses (65, 66). Similarly, ultrasonic cavitation effects produce free radicals, which act as inducing factors that stimulate the release of DAMPs (67). DAMPs then activate inflammatory reaction pathways, lymphocytes, monocytes and macrophages release IL-1 and IL-18 inflammatory regulating cell factors, promoting tumor antigens presentation for induction of T cells adaptive responses, which improves tumor immune escape (68) (**Figure 2**).

Ca2+ plays a key role in cell integrity, membrane encapsulation, and intercellular signaling. Ultrasonic cavitation can increase intracellular Ca2+ levels by inducing adjacent intracellular Ca2+ increase *via* intercellular signaling to neighboring cells (70). Beekers et al. (71) showed that an MB oscillation amplitude between 0.75 μm and 1 μm could maintain stable cell viability, but increasing the amplitude oscillation to greater than 1 μm would cause dramatic fluctuation in Ca2+ concentration. They also showed that contact between adjacent cells was opened when irreversible Ca2+ fluctuations were caused by ultrasound-induced MB oscillation, suggesting that the opening of intercellular contact is a biological response caused by elevated Ca2+ levels; a mechanism that also facilitates drug passage through the BBB (72). Thus, increasing oscillation amplitude increases the degree of pore damage and decreases the ability of cell membranes to reseal. This leads to activation of voltage-sensitive Ca2+ channel, through which extracellular Ca2+ flows into the cell, causing drastic Ca2+ fluctuations and Ca2+ overload.

Some endonucleases responsible for DNA fragments are Ca2+-dependent, and once Ca2+ concentration increases, the enzyme is activated and degrades DNA to induce apoptosis (73). Similarly, Shi et al. (74) found that Ca2+-dependent endonucleases and protease activation could lead to the apoptosis of hepatocellular carcinoma cells, SMMC-7221, by opening their mitochondrial pores and increasing membrane permeability. The ceramide signaling pathway instigated by ECs injury has a significant role in controlling cancer cell demise (14). Al-mahrouki et al. (75) confirmed that UTMD-induced ceramide accumulation was caused by the downregulation of UDP glycosyltransferase-8. The anti-apoptotic function of UDP glycosyltransferase-8 was achieved by disrupting the ceramide signaling pathway and converting ceramide to galactose ceramide.

**Figure 3:**
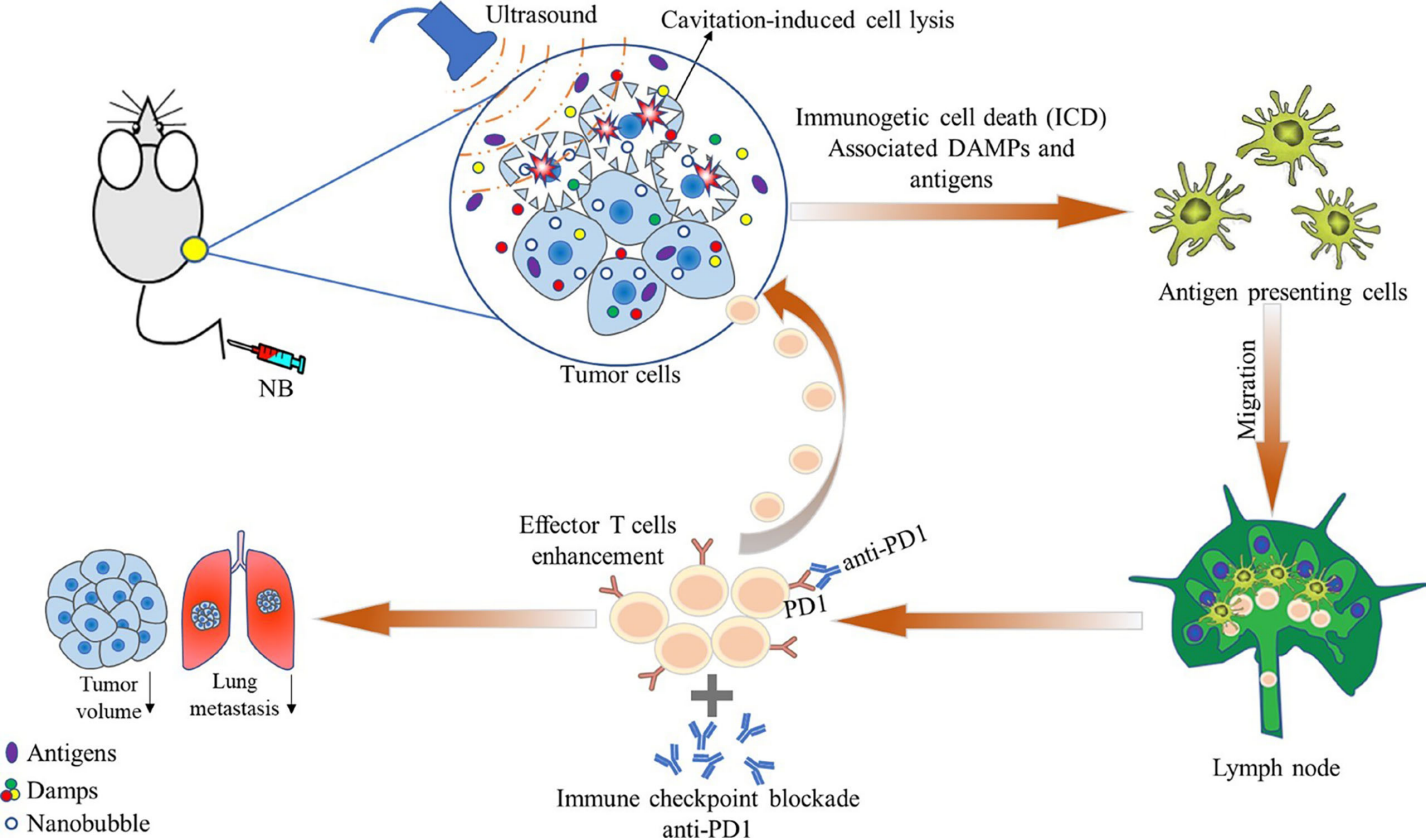
Schematic illustration showing the effects of ultrasound-stimulated nanobubbles (USNBs) on the mouse tumor model. USNBs can induce tumor cell necrosis, which can release immunogenic substances, further activate innate and adaptive immune cells, and finally activate CD8+ T cells. This leads to systemic anti-tumor immunity, enhancing the efficacy of anti-PD1 therapy and promoting immune memory. Reprinted (adapted) with permission from ref (18). copyright ^©^ 2022 Hu, He, Wang, Zhao, Fang, Dong, Chen, Zhang, Zhang, Wang, Tan, Wang, Zi, Liu, Liang, Guo, Ou.

In a study by Hu et al. (18), the authors compared the antitumor effect of ultrasound-stimulated nanobubbles alone and in combination with an anti-programmed cell death protein 1 (aPD-1) in RM1 (prostate cancer), MC38 (colon cancer), and B16 (melanoma) xenograft mouse models. They found that ultrasound-stimulated nanobubbles combined with aPD-1 induced tumor cell necrosis, significantly increased the release of DAMP and tumor antigen presentation, and promoted the invasion and antitumor activity of CD8+ T cells (**Figure 3**). Thus, with this strategy, immunogenicity can be improved by remodeling the tumor immune microenvironment and sensitizing poorly immunogenic solid tumors to aPD-1 treatment.

## Application of UTMD in Tumor Immunotherapy

### UTMD-Mediated Tumor Vaccine

Therapeutic cancer vaccines are an active immunotherapy approach to induce durable antitumor immunity. These include tumor cell vaccines, dendritic cell (DC) vaccines, viral vector vaccines, and molecular vaccines composed of peptides, DNA or RNA (76, 77). DC vaccines are most commonly used in tumor immunotherapy due to their high antigen presentation effect. High-efficiency antigen-presenting cells DC load tumor-associated antigens into the body and activate T cells. Some of them are activated and proliferate into cytotoxic T lymphocytes, causing a strong immune response, while some become long-term memory T cells, producing immune memory. Since the approval of PROVENGE (Sipuleucel-T), the first DC vaccine, to treat advanced resistant prostate cancer by the Food and Drug Administration (FDA) in 2010 (78), several cancer vaccines have been developed against melanoma (NeoVax) and non-small cell lung cancer. Some of them have shown promising benefits and are being further tested in clinical trial settings. However, due to the low efficiency of traditional antigen infusion methods, inducing an effective immune effect with tumor vaccines has been very challenging (79). Therefore, the key of current research is to deliver an adequate concentration of antigens to DC for effective activation of antitumor immunity and preventing the degradation of antigen.

Suzuki et al. (23) delivered antigen into DC using an ultrasonic approach combined with foam liposomes, which was found to act similarly to ultrasonic MBs. The antigen passed through the transient pore produced by cavitation effects without entering the cytoplasm of DC through the endocytosis pathway. This delivery method directly enabled the model antigen (ovalbumin, OVA) to enter the major histocompatibility complex (MHC) class I presentation pathway and activated exogenous antigen-specific cytotoxic T lymphocytes. Further, Oda et al. (22) demonstrated that UTMD combined with immunotherapy could deliver tumor extracted antigen to DC and reduce the incidence of pulmonary metastasis of melanoma by four times. These indicate that bubble liposomes combined with ultrasound could be an effective method to transport antigen to DC. Additionally, studies have shown that immersion of nano-cavitated nuclei with model antigen (OVA) in hydrogel and exposure to ultrasound could significantly increase the transdermal delivery dose and enhance vaccine model antigen penetration, which was associated with highly-specific effects on anti-OVA IgG antibody levels in mice. These results indicate that ultrasound combined with nano cavitation nucleus has potential prospects in adjuvant percutaneous needle-free tumor vaccine vaccination (80). Meng et al. (81) designed an injectable self-healing hydrogel system loaded with nano-vaccines which could be converted into a sol state after ultrasonic treatment, allowing the release of the vaccine and then self-healing into a gel. Thus, multiple ultrasound treatments can repeatedly release nano-vaccines and produce effective antitumor immune responses, allowing one-time ultrasonic mediated inoculation and multiple effective treatments.

### UTMD-Mediated Antibody Immunotherapy

Monoclonal antibodies are among the most successful and important strategies for treating patients with hematological malignancies and solid tumors. Due to rapid developments in the field of immunology and protein engineering, monoclonal antibodies are currently the fastest-growing type of immunotherapy (82, 83). Monoclonal antibodies exert their tumor-killing effects *via* complement-mediated cytotoxicity and antibody-dependent cytotoxicity, while immune checkpoint inhibitors exert their antitumor effects by blocking immunosuppressive signals. In addition, antibody-coupled drugs can specifically bind to tumor surface antigens, releasing drugs that kill tumor cells and activate the immune system. UTMD can assist in the targeting and releasing of antibodies to target tissues, increase treatment efficiency and reduce systemic toxicity.

**Figure 4:**
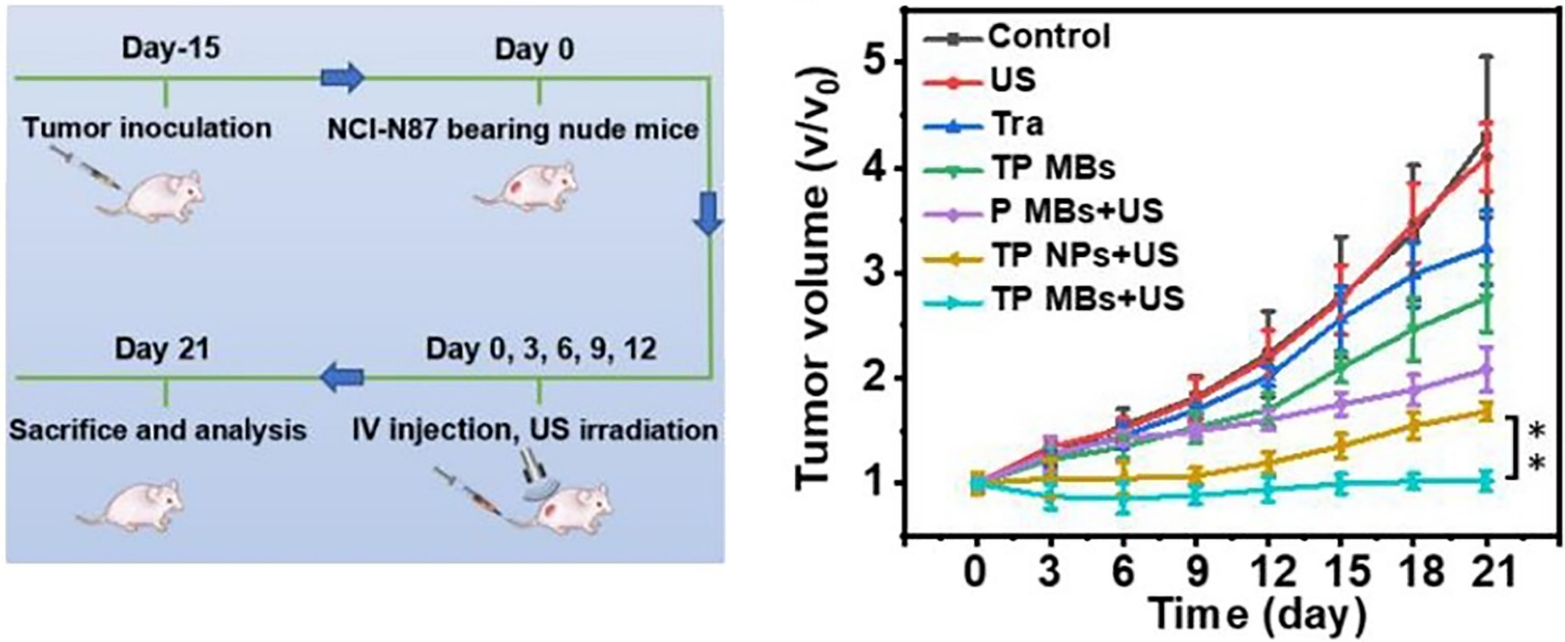
Ultrasound microbubbles mediated sonosensitizer and trastuzumab (TP MBs) treatment significantly inhibited the proliferation of tumor cells. The TP MBs + US group showed the best therapeutic effect with almost no tumor volume change for 21 days. Reprinted (adapted) with permission from ref (20). copyright ^©^ 2022 American Chemical Society. **p<0.01.

To investigate the therapeutic effect of UTMD-mediated chemotherapy drugs combined with monoclonal antibodies in multiple myeloma tumor stem cell transplantation mouse models, Shi et al. (84) developed lipoid MBs loaded with epirubicin and combined them with anti-ABCG2 monoclonal antibody. They found that, compared with no ultrasound irradiation, the combined approach could effectively inhibit the growth of multiple myeloma, prolong the survival time of mice, and alleviate the symptoms of multiple myeloma. In addition, the approach was more targeted than epirubicin therapy alone and was associated with reduced cardiac toxicity in mice models. Sun et al. (20) constructed an ultrasonic MB loaded with trastuzumab coupled with acoustic sensitizer nanoparticles. They found that the delivery and treatment efficiency with nanoparticles was improved with UTMD and successfully inhibited the proliferation of tumor cells, achieving a targeted combination of sonodynamic therapy and antibody therapy with nanoparticles for treating HER2-positive gastric cancer (**Figure 4**).

UTMD can increase antigen release, heat shock protein expression, calreticulin levels and pro-phagocytic signals, affecting the tumor microenvironment and comprehensively stimulating tumor immunity (85, 86). In a study by Ye et al. (21), the authors evaluated the ability of UTMD to enhance the targeted accumulation of aPD-L1 in the brain stem. They used UTMD to deliver the study drug to a brain of a mouse glioma model *via* the intranasal route. Anti-programmed cell death-ligand1 antibody (aPD-L1) was alternately dropped through the nostril, followed by MBs injected through the tail vein and an immediate head ultrasonic irradiation. Their results showed that, compared with intranasal administration alone, UTMD enhanced the targeted accumulation of aPD-L1 in the brain stem after intranasal administration and improved the penetration depth and transmission efficiency of aPD-L1 in the brain parenchyma. The accumulation rates in mice with and without tumor were similar, suggesting that the effect of UTMD-mediated intranasal brain drug delivery was not affected by the tumor microenvironment.

### UTMD Mediated Gene Therapy

Gene therapy methods include viral vector transfection and non-viral transfection. Retrovirus and adenovirus transfection methods have systemic toxicity, insertion mutation and other problems (87, 88). In contrast, non-viral chemical and physical transfection methods seem safer, have lesser toxicity and are more specific than viral vector transfection methods (89). Thus, UTMD-mediated gene transfer is one of the most promising non-viral physical delivery methods.

The cavitation effect of ultrasound produces instantaneous pores on the cell membrane. Different ultrasonic peak negative pressures can create instantaneous pores of different sizes and mediate the entry of plasmids through these pores into cells (90–92). After ultrasonic irradiation, the membrane regains its integrity, seals all the pores, and traps the material delivered inside the cell (93). Plasmid DNA uptake induced by UTMD is a rapid, multi-mechanistic process that is not limited to the site of MB attachment (94). The continuity and fluidity of membrane lipid bilayer and the interaction between membrane and cytoskeleton may also be associated with plasmid DNA uptake (46). Due to low toxicity, low immunogenicity and high targeting efficiency, UTMD-mediated gene transfer has shown great application prospects in clinical gene therapy, and has been successfully applied in various tissues and organs, including muscles (95), kidneys (96), liver (97), parotid gland (98) and retina (99), and.

Zhang et al. (24) introduced plasmids into tumor cells using UTMD of different MB sizes. They observed that the transfection rate of the larger MBs (4.23 ± 2.27 μm) was significantly higher than those of smaller MBs (1.27 ± 0.89 μm), and 29.7% of the tumor cells were transfected into the DNA plasmid. Further, after 48 h, the gene expression in tumors using UTMD with larger MBs was more than tripled that of smaller MBs, and had greater infiltration of CD8 T cells and F4/80 macrophages. Dong et al. (25) investigated the effectiveness of *in vivo* UTMD (ultrasonic peak negative pressure: 5.5 MPa) delivery of pre-miRNAs plasmids, and observed that UTMD could effectively inhibit subcutaneous tumor growth in a mouse liver cancer model. They also found that the plasmid delivery efficiency and cell viability were positively correlated with peak negative ultrasound pressure. In a study by Ilovitsh et al. (26), the authors found that combining UTMD with intraperitoneal administration of checkpoint inhibition and IFN-β plasmid transfection could significantly reduce tumor volume and enhance T cell infiltration by recruiting effective local and distant tumor site immune cells.

### UTMD-Mediated Adoptive Cell Immunotherapy

Adoptive cell therapy is a passive immunotherapy method in which a large number of amplified and activated immune cells after *in vitro* genetic engineering or screening activation are transfused back into the patient to enhance immune responses in the tumor microenvironment and directly or indirectly achieve tumor-killing effects (100). Unlike T cells and B cells, natural killer (NK) cells can express high levels of effector molecules with cytotoxicity, including perforin and granase B, making NK cells the most widely used in adoptive immunotherapy (100, 101). However, the antitumor functions of NK cells in solid tumors are still unclear. The main reason is that the injection of NK cells cannot fully home at the tumor site, leading to a low number of NK cells targeting tumor cells and inadequate effective immune responses. Thus, improving the homing of NK cells at tumor sites could improve therapeutic outcomes (102).

Studies have shown that the stable cavitation effects produced by low-intensity focused ultrasound combined with MB therapy could promote the homing of various cells, including CD8+ cytotoxic T lymphocytes, dendritic cells, NK cells, neutrophils and macrophages, and could induce effective immune responses by disrupting the tight junctions of endothelial cells, increasing vesicular transport and changing ECs membrane proteins (103–105). Thus, UTMD has the potential to provide effective targeted delivery of adoptive cells to tumor lesions.

Alkins et al. (27) demonstrated that MRI-guided low-intensity focused ultrasound combined with MBs (peak sound pressure: 0.33 MPa) could target the implantation of NK-92 cells specifically expressing HER2 into the brain of nude mice before the BBB is destroyed, thereby increasing the high number of effector cells at the metastatic brain tumor site. Intravenous injection of HER2-specific NK-92-scFv (FRP5) zeta cell line, in early tumor developmental stages before BBB disruption, using MRI-guided focused-ultrasound combined with MB local irradiation to the tumor inhibited tumor growth in metastatic breast cancer model by amplifying HER2. This led to a significant reduction in the mean tumor volume, measured on the 28th day, and prolonged the survival of the mice. In a study by Yang et al. (102), the authors investigated the tumor-shrinking efficacy of UTMD combined with NK-92MI versus NK-92MI alone. They observed that although the addition of UTMD demonstrated an accumulation of adoptive NK-92MI cells from blood vessels to the tumor site, the difference in tumor volume reduction between the two groups was not statistically significant. The reason for such observation could be related to an insufficient number of NK cells entering the tumor, leading to low tumor-killing efficacy. Therefore, to improve the transfer efficiency of NK-92MI cells in the treatment of solid tumors, it is still necessary to further optimize the MB dose, ultrasound irradiation time and treatment frequency in future studies.

Sta Maria et al. (28) treated xenograft tumors of human colorectal adenocarcinoma mice with low dose focused ultrasound with MBs (peak sound pressure: 0.50 MPa) and injected MBs plus NK cells into the mouse tail vein. They observed that within 24 h of treatment, the aggregation of NK cells in the 0.5 MPa low-dose ultrasound group was significantly greater than in the non-low-dose ultrasound group, while no NK cell aggregation was observed in the 0.25 MPa low-dose ultrasound group. These observations suggest that sound pressure could be an important factor affecting the local homing of NK cells to tumor sites and the systemic effects of NK cells.

## Discussion, Conclusion, and Outlook

Immunotherapy has enormous potential in cancer treatment and has offered patients with advanced malignant tumors new and promising treatment options. The recent combined application of UTMD with tumor immunotherapy has shown great potential in amplifying immunotherapy outcomes as nanometer MB can extend the time for payload drug activities or gene foam half-life, thereby increasing the bioavailability, specificity, and specificity durability of immunotherapy to the tumor site, whilst decreasing systemic toxicity. However, the optimal dose of MB, time for ultrasonic irradiation, and treatment frequency are still undetermined and should be further explored. Different cancer types and individual genetic background need to be taken into account in UTMD combined immunotherapy (106). At present, UTMD-mediated tumor immunotherapy is mainly in an investigational stage in *in vitro* experiments. Many potential mechanisms of the biological effects of vascular ECs induced by UTMD have not yet been deeply explored, and further clarifications on their underlying mechanisms are still needed to better assist tumor immunotherapy.

In the future, the cross fusion between tumor immunotherapy and other therapeutic methods could further improve the outcomes of tumor immunotherapy. The prospects of combining new technologies and methods with immunotherapy to safely and effectively destroy tumor cells and ultimately achieve the goal of a non-toxic and lasting cure remain promising.

## Author Contributions

YH, HW, JH, and WL contributed to conception and design of the study. JS wrote the first draft of the manuscript. XW, YH, JH, and WL wrote sections of the manuscript. All authors contributed to manuscript revision, read, and approved the submitted version.

## Conflict of Interest

The authors declare that the research was conducted in the absence of any commercial or financial relationships that could be construed as a potential conflict of interest.

## Publisher’s Note

All claims expressed in this article are solely those of the authors and do not necessarily represent those of their affiliated organizations, or those of the publisher, the editors and the reviewers. Any product that may be evaluated in this article, or claim that may be made by its manufacturer, is not guaranteed or endorsed by the publisher.
